# Combination of Cerebral Computed Tomography and Simplified Cardiac Arrest Hospital Prognosis (sCAHP) Score for Predicting Neurological Recovery in Cardiac Arrest Survivors

**DOI:** 10.31083/j.rcm2401025

**Published:** 2023-01-12

**Authors:** Sih-Shiang Huang, Yu-Tzu Tien, Hsin-Yu Lee, Hooi-Nee Ong, Chien-Hua Huang, Wei-Ting Chen, Wen-Jone Chen, Wei-Tien Chang, Min-Shan Tsai

**Affiliations:** ^1^Department of Emergency Medicine, National Taiwan University Medical College and Hospital, 100 Taipei, Taiwan; ^2^Department of Internal Medicine (Cardiology division), National Taiwan University Medical College and Hospital, 100 Taipei, Taiwan; ^3^Department of Internal Medicine, Min-Sheng General Hospital, 330 Taoyuan, Taiwan

**Keywords:** severity score, cardiac arrest, neuroprognostication, gray-to-white ratio, cerebral CT

## Abstract

**Background:**

Cerebral computed tomography (CT) and various severity scoring systems have been developed for the early prediction of the neurological outcomes of cardiac arrest survivors. However, few studies have combined these approaches. Therefore, we evaluated the value of the combination of cerebral CT and severity score for neuroprognostication.

**Methods:**

This single-center, retrospective observational study included consecutive patients surviving nontraumatic cardiac arrest (January 2016 and December 2020). Gray-to-white ratio (GWR), third and fourth ventricle characteristics, and medial temporal lobe atrophy scores were evaluated on noncontrast cerebral CT. Simplified cardiac arrest hospital prognosis (sCAHP) score was calculated for severity assessment. The associations between the CT characteristics, sCAHP score and neurological outcomes were analyzed.

**Results:**

This study enrolled 559 patients. Of them, 194 (34.7%) were discharged with favorable neurological outcomes. Patients with favorable neurological outcome had a higher GWR (1.37 vs 1.25, *p *< 0.001), area of fourth ventricle (461 vs 413 ${\mathrm{mm}}^{2}$, *p *< 0.001), anteroposterior diameter of fourth ventricle (0.95 vs 0.86 cm , *p *< 0.001) and a lower sCAHP score (146 vs 190, *p *< 0.001) than those with poor recovery. Patients with higher sCAHP score had lower GWR (*p *trend < 0.001), area of fourth ventricle (*p *trend = 0.019) and anteroposterior diameter of fourth ventricle (*p *trend = 0.014). The predictive ability by using area under receiver operating characteristic curve (AUC) for the combination of sCAHP score and GWR was significantly higher than that calculated for sCAHP (0.86 vs 0.76, *p *< 0.001) or GWR (0.86 vs 0.81, *p* = 0.001) alone.

**Conclusions:**

The combination of GWR and sCAHP score can be used to effectively predict the neurological outcomes of cardiac arrest survivors and thus ensure timely intervention for those at high risk of poor recovery.

## 1. Introduction

Sudden cardiac arrest remains a major challenge in clinical practice and accounts for more than 356,000 and 290,000 annual cases of out-of-hospital cardiac arrest (OHCA) and in-hospital cardiac arrest (IHCA), respectively, in the United States [1, 2]. In Taiwan, data from the National Health Insurance Administration indicate an OHCA incidence of 51.1 per 100,000 individuals [3]. Owing to hypoxic–ischemic brain injury after cardiac arrest, cognitive problems are common in cardiac arrest survivors [4]. Early and accurate prediction of the neurological outcomes of cardiac arrest survivors is crucial for determining the extent of medical resources required and for avoiding the inappropriate withdrawal of life-sustaining treatment for those with potential for favorable neurological recovery [5, 6]. In addition, neurological recovery may be delayed after therapeutic temperature management (TTM) because of the use of sedatives [7, 8]. Therefore, several tools have been developed for neuroprognostication for cardiac arrest survivors; these include brain imaging modalities, severity scores, electrophysiological monitoring data, and biomarkers [9].

Gray-to-white ratio (GWR), the ratio of gray matter to white matter on cerebral computed tomography (CT), has been explored as a marker of the severity of hypoxic–ischemic encephalopathy among cardiac arrest survivors [10, 11, 12, 13]. Moreover, some ventricular characteristics detected on cerebral CT, such as the area of lateral ventricles, ventricle-to-brain ratio (VBR), anteroposterior diameters, and size of the third and fourth ventricles, have been used as predictive markers [14, 15]. Medial temporal lobe atrophy (MTLA) scores help predict cognitive function [16], and the extent of brain atrophy has been recently used to predict the cognitive outcomes of OHCA survivors [17, 18]. Some studies focused on using cerebral magnetic resonance imaging (MRI) for neurological outcome prediction in cardiac arrest survivors and showed prominent result [17, 18, 19, 20]; however, most of the relevant studies had small sample sizes and the group of patients who had MRI-incompatible internal cardiac defibrillators would be excluded. In addition to brain image, several other electrophysiological monitoring and clinical scoring systems have been established for illness severity and prognostication. The somatosensory evoked potentials (SSEP) is now widely accepted as one of the multimodal approach tools for functional outcome prediction in cardiac arrest survivors. Some studies concluded that it may be the earliest predictor for favorable neurological outcomes; however, the self-fulfilling prophecy is still a major concern [21, 22]. The simplified cardiac arrest hospital prognosis (sCAHP) score is a validated tool for the early prediction of poor neurological outcomes at hospital discharge [23, 24]. sCAHP scores are advantageous over CAHP score in that they do not include a parameter corresponding to no-flow time, which is difficult to estimate for unwitnessed OHCA. GWR is one of the eight factors of the post-Cardiac Arrest Syndrome for Therapeutic Hypothermia (CAST) score for the early prediction of neurological outcomes after cardiac arrest [25]. A revised CAST score was proposed in which the calculation is simplified through the deletion of three of the eight CAST factors: GWR, albumin level, and hemoglobin level [26]. In a single-center retrospective study, the two scores, with and without GWR, were compared, but no substantial differences were noted in the prognostic value of the two scores [27]. Limited evidence is available to indicate whether GWR still plays a crucial role in overall interpretation in addition to severity score for the neuroprognostication. Furthermore, the correlations between cerebral CT parameters and severity scores remain unclear. Therefore, we investigated whether predictive markers from cerebral CT are correlated with arrest severity scores. In addition, we evaluated the benefits of combining neuroimaging data with severity scores for predicting the neurological outcomes of cardiac arrest survivors.

## 2. Materials and Methods

### 2.1 Study Design and Patients

**Fig. 1.**
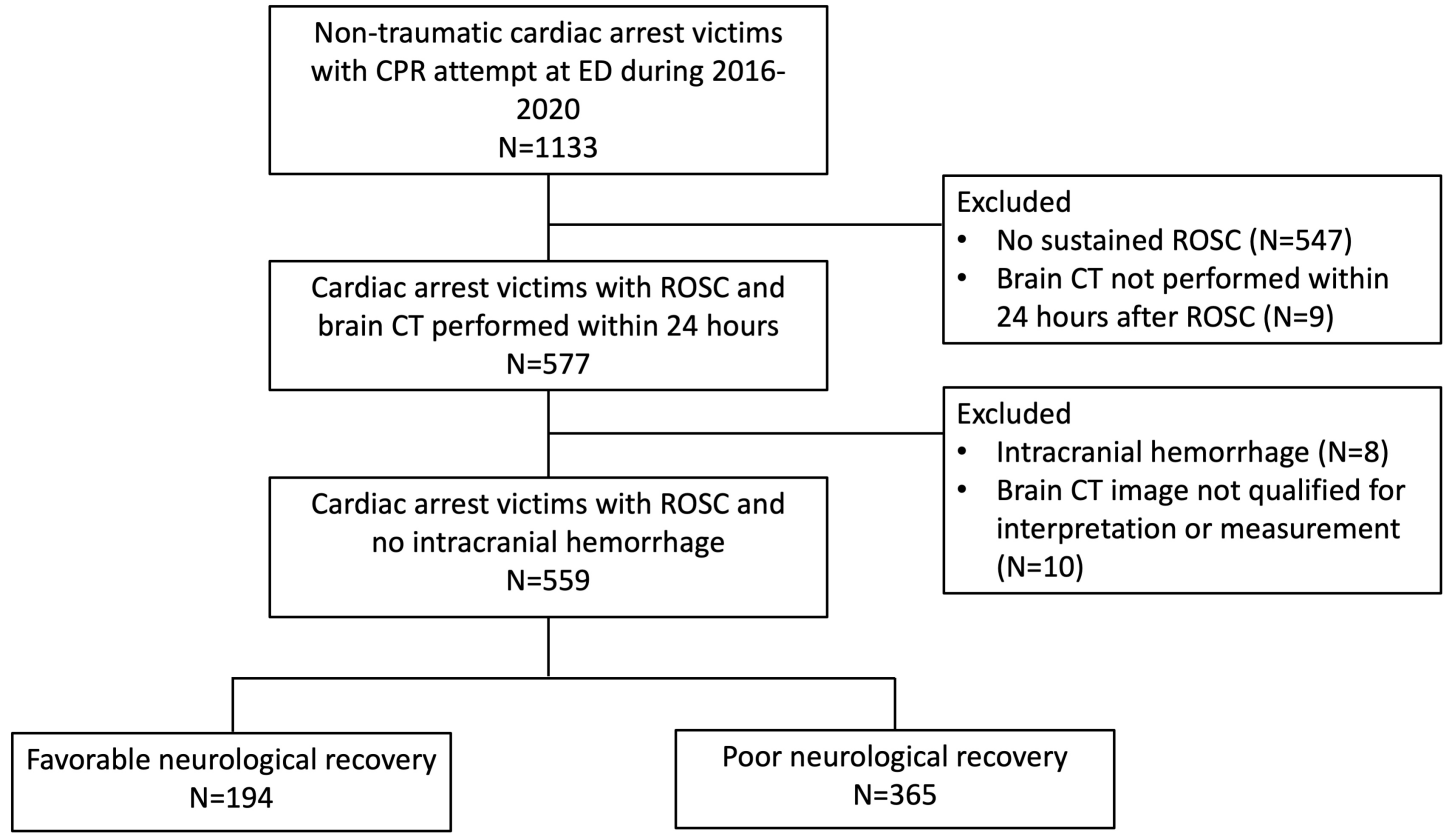
**Flowchart of patient enrollment**. CPR, cardiopulmonary resuscitation; CT, computed tomography; ED, emergency department; ROSC, return of spontaneous circulation.

The retrospective observational study, approved by the Institutional Review Boards of National Taiwan University Hospital (NTUH) (202112205RINB), enrolled 1133 non-traumatic adult cardiac arrest patients between January 2016 to December 2020 at a single tertiary medical center in Taipei, Taiwan, and the requirement of informed consent was waived. After excluding patients without sustained return of spontaneous circulation (ROSC) (n = 547) and without cerebral CT within 24 h after ROSC, there were 577 nontraumatic adult cardiac arrest survivors who underwent cerebral CT within 24 h after ROSC. Patients whose cerebral CT images were unsuitable for interpretation or measurement (n = 10) and those whose cerebral CT findings revealed intracranial hemorrhage (n = 8) were excluded. Finally, 559 patients were included. Of them, 194 patients (34.7%) were discharged with favorable neurological outcomes, defined as a score of 1 or 2 on the Glasgow–Pittsburgh cerebral performance category (CPC) scale, and constituted the favorable outcome group. The remaining 365 patients exhibited poor neurological recovery (CPC score of 3–5) at discharge and constituted the poor outcome group (Fig. 1).

### 2.2 Data Collection

The patients’ underlying characteristics, disease severity, cardiac arrest events, and postarrest care were collected from medical records by using a predesigned questionnaire based on the Utstein Style [28]. OHCA was defined as the absence of circulation outside the hospital, and IHCA was defined as the absence of circulation after triage. Transferred patients with cardiac arrest were defined as those patients who were successfully resuscitated at another hospital and then transferred to NTUH. Prehospital ROSC was defined as the return of the heartbeat and pulse in patients with OHCA before their arrival at the hospital, as evident from their emergency medical service records. Ischemic heart disease, heart failure, structural heart disease, or arrhythmia without considerable electrolyte imbalance was regarded as the primary cause of cardiac arrest. The causes of cardiac arrest were determined by responsible primary care physicians who were blinded to the group allocation. To evaluate cardiac arrest severity at ROSC, the sCAHP score was calculated [23]. The TTM protocol at NTUH includes reducing the patient’s body temperature to a target temperature (33 °C) within 6 h after ROSC, maintaining the target temperature for 24 h, rewarming the patient by gradually increasing (0.25 °C/h; using BD Arctic Sun 5000 Temperature Management System (Franklin Lakes, NJ, USA) with automated feedback) the body temperature to 36 °C, and maintaining the body temperature at <36.5 °C for 24 h after complete rewarming. The highest acute physiology and chronic health evaluation (APACHE) II score within 24 h after ROSC was recorded.

### 2.3 Cerebral CT Measurements

Noncontrast cerebral CT images obtained using a 64-slice CT scanner (5-mm slice; LightSpeed, GE Healthcare, Chicago, IL, USA) were analyzed by two investigators (SSH and HYL) who were blinded to the final neurological outcomes. The investigators measured the Hounsfield unit (HU) values of the putamen and corpus callosum at the level of the basal ganglia [13] as well as the anteroposterior diameter of the fourth ventricle. In addition, the patients’ MTLA scores were obtained from the National Taiwan University hospital’s picture archiving and communication system [29]. The MTLA score is a radiographic evaluation of brain atrophy. Abnormal MTLA scores were defined as scores of ≥2 in patients aged <75 years and scores of ≥3 in patients aged ≥75 years. GWR was calculated as the ratio of the average HU value of the bilateral putamen to that of the bilateral corpus callosum. To determine the areas of the entire brain, lateral ventricles, and third and fourth ventricles, MIPAV (http://mipav.cit.nih.gov/ 11.0.7, Center for Information Technology, National Institutes of Health at Bethesda, MD, USA) was used. The region of interest was drawn adjacent to the target structure to calculate the area in square millimeters [30]. VBR was calculated as the ratio of the total area of the two lateral ventricles to that of the entire brain.

### 2.4 Statistical Analysis

Categorical variables were compared using the chi-square or Fisher’s exact test and are expressed in terms of numbers and percentages. Continuous variables were compared using the Mann-Whitney U test and are expressed in terms of medians and interquartile ranges. *p* values for trends (*p *trend) were calculated to evaluate the differences in the CT characteristics of the aforementioned groups. Receiver operating characteristic (ROC) curves were plotted, and the areas under the ROC curves (AUCs) were calculated to evaluate the performance of GWR in predicting neurological outcomes. The DeLong test was performed to compare the ROC curves. Statistical significance was set at* p *< 0.05. All data were analyzed using R 4.1.1 (R Foundation for Statistical Computing, Vienna, Austria).

## 3. Results

The median age of the study cohort was 66.4 (55.0–77.3) years, and 395 (70.7%) of the patients were men. The numbers of patients with OHCA, patients with IHCA, and transferred patients were 348 (62.2%), 162 (29.0%), and 49 (8.8%), respectively. Most of the patients (482; 86.2%) had witnessed collapse, and 35.1% had initial shockable rhythms.

Table 1 summarizes the demographic characteristics of the cardiac arrest survivors. The favorable outcome group was younger than the poor outcome group (61.3 vs 68.7 years, *p *< 0.001). Regarding their underlying characteristics, no marked differences were noted between the groups except in malignancy, which was more prevalent in the poor outcome group than in the favorable outcome group (28.2 vs 13.4%, *p *< 0.001). Regarding cardiac events, compared with those of the poor outcome group, the favorable outcome group had more prehospital ROSC (6.8 vs 25.8%, *p *< 0.001), and fewer total cardiopulmonary resuscitation (CPR) duration (15.2 vs 25.4 min, *p *< 0.001), CPR >10 min (95.3 vs 86.6%, *p *< 0.001), repeated CPR (23.6 vs 11.9%, *p* = 0.001), or Epinephrine ≥3 mg during resuscitation (50.7 vs 23.2%, *p *< 0.001). More patients in the favorable neurological outcome groups were classified as low severity in the sCAHP score (52.2 vs 15.9%, *p *< 0.001). Similarly, there was higher proportion of patients with high severity in the poor outcome group than in the favorable outcome group (40.0 vs 9.3%, *p *< 0.001). Regarding postarrest care, compared with the poor outcome group, the favorable outcome group had higher systolic blood pressure (132 vs 114 mmHg, *p *< 0.001), diastolic blood pressure (79.0 vs 64.5 mmHg, *p *< 0.001), hemoglobin levels (14.3 vs 12.0 g/dL, *p *< 0.001), and pH (7.20 vs 7.08, *p *< 0.001). Compared with the favorable outcome group, the poor outcome group exhibited higher APACHE II scores (35 vs 30, *p *< 0.001), high-sensitivity troponin-T levels (53.4 vs 25.8 ng/L, *p *< 0.001), and lactic acid levels (9.84 vs 8.04 mmol/L, *p *< 0.001).

**Table 1.** **Baseline characteristics of studied patients**.

			All patients	Favorable outcome	Poor outcome	*p*-value
			(n = 559)	(n = 194)	(n = 365)	
Male, n (%)			395 (70.7)	143 (73.7)	252 (69.0)	0.291
Age ≥65 years			308 (55.1)	80 (41.2)	228 (62.5)	<0.001
Age, years, median (IQR)			66.4 (55.0–77.3)	61.3 (51.1–72.1)	68.7 (57.8–79.4)	<0.001
Underlying characteristics, n(%)						
	Hypertension		316 (56.5)	109 (56.2)	207 (56.7)	0.976
	Diabetes mellitus		177 (31.7)	54 (27.8)	123 (33.7)	0.186
	Hyperlipidemia		64 (11.4)	26 (13.4)	38 (10.4)	0.359
	Coronary artery disease		190 (34.0)	57 (29.4)	133 (36.4)	0.113
	Heart failure		54 (9.7)	18 (9.3)	36 (9.9)	0.942
	Valvular heart disease		18 (3.2)	7 (3.6)	11 (3.0)	0.899
	Arrhythmia		75 (13.4)	32 (16.5)	43 (11.8)	0.154
	COPD/Asthma		53 (9.5)	17 (8.8)	36 (9.9)	0.786
	Post-tracheostomy		11 (2.0)	2 (1.0)	9 (2.5)	0.399
	Renal disease		49 (8.8)	18 (9.3)	31 (8.5)	0.877
	ESRD		61 (10.9)	22 (11.3)	39 (10.7)	0.925
	Liver cirrhosis		11 (2.0)	1 (0.5)	10 (2.7)	0.138
	CVA		50 (8.9)	15 (7.7)	35 (9.6)	0.564
	Dementia		19 (3.4)	4 (2.1)	15 (4.1)	0.305
	Bedridden		21 (3.8)	5 (2.6)	16 (4.4)	0.403
	Malignancy		129 (23.1)	26 (13.4)	103 (28.2)	<0.001
Cardiac arrest events, n(%)						
	Source					0.561
	OHCA		348 (62.2)	123 (63.4)	225 (61.6)	
	IHCA		162 (29.0)	59 (30.4)	103 (28.2)	
	Transfer		49 (8.8)	12 (6.2)	37 (10.1)	
	Witnessed collapse		482 (86.2)	179 (92.3)	303 (83.0)	0.004
	Initial shockable rhythm		196 (35.1)	104 (53.6)	92 (25.2)	<0.001
	Pre-hospital ROSC		74 (13.2)	50 (25.8)	24 (6.8)	<0.001
	Total CPR duration (min)		21.9 ± 7.3	15.2 ± 6.0	25.4 ± 7.6	<0.001
		CPR >10 min	516 (92.3)	168 (86.6)	348 (95.3)	<0.001
	Repeated CPR		109 (19.5)	23 (11.9)	86 (23.6)	0.001
	Epinephrine ≥3 mg		230 (41.1)	45 (23.2)	185 (50.7)	<0.001
	Cardiogenic arrest		302 (54.0)	133 (68.6)	169 (46.3)	<0.001
sCAHP severity						
	Low (<150)		159 (28.4)	101 (52.1)	58 (15.9)	<0.001
	Moderate (150–200)		212 (37.9)	67 (34.5)	145 (39.7)	0.250
	High (>200)		164 (29.3)	18 (9.3)	146 (40.0)	<0.001
Post-arrest care, median (IQR)						
	ROSC SBP, mmHg		119 (99–154)	132 (104–161)	114 (84.0–149)	<0.001
	ROSC DBP, mmHg		69 (54.0–88.5)	79 (62.0–94.5)	64.5 (50.0–84.0)	<0.001
	TTM		210 (37.6)	66 (34.0)	144 (39.5)	0.242
	APACHE II score		34 (27.5–39.0)	30 (19.5–36.0)	35 (31–40)	<0.001
	Hemoglobin, g/dL		12.6 (10.3–15.1)	14.3 (11.0–16.0)	12.0 (9.7–14.4)	<0.001
	Troponin-T, ng/L		36.4 (16.0–116)	25.8 (14.3–71.1)	53.4 (20.1–142)	<0.001
	Lactic acid, mmol/L		9.38 (6.23–12.3)	8.04 (5.34–11.4)	9.84 (6.69–12.6)	<0.001
	pH value		7.12 (7.00–7.25)	7.20 (7.05–7.29)	7.08 (6.98–7.20)	<0.001
	${\mathrm{HCO}}_{3}$, mmol/L		19.0 (15.4–22.9)	19.2 (15.4–22.8)	18.9 (15.4–23.0)	0.853

Data presented as no. (%) or as median (IQR). COPD, chronic obstructive pulmonary disease; CPR, cardiopulmonary resuscitation; CVA, cerebrovascular accident; DBP, diastolic blood pressure; ESRD, end stage renal disease; IHCA, in-hospital cardiac arrest; IQR, interquartile range; OHCA, out-of-hospital cardiac arrest; ROSC, return of spontaneous circulation; SBP, systolic blood pressure; sCAHP, simplified cardiac arrest hospital prognosis; TTM, therapeutic temperature management; APACHE, acute physiology and chronic health evaluation.

Table 2 summarizes the cerebral CT characteristics of each group. The test–retest reliability of the neuroimaging measurements was characterized by excellent intraclass and interrater correlation coefficients of 0.960 and 0.909, respectively. Compared with the poor outcome group, the favorable outcome group had a significantly higher GWR (1.37 vs 1.25, *p *< 0.001) and anteroposterior diameter of the fourth ventricle (0.95 vs 0.86 cm, *p *< 0.001). The fourth ventricle area was larger in the favorable outcome group than in the poor outcome group (461 vs 413 ${\mathrm{mm}}^{2}$, *p *< 0.001). However, no significant differences were noted between the groups in lateral ventricle area, third ventricle area, VBR or abnormal MTLA score. The cerebral CT characteristics were compared between groups stratified by sCAHP score (Table 3). Significantly lower GWR (*p* trend < 0.001), lower area (*p* trend = 0.019) and anteroposterior diameter (*p* trend = 0.014) of the fourth ventricle were associated with higher illness severity.

**Table 2.** **Characteristics of cerebral computed tomography between groups**.

	All patients	Favorable outcome	Poor outcome	*p*-value
	(n = 559)	(n = 194)	(n = 365)	
GWR, Median (IQR)	1.29 (1.21–1.37)	1.37 (1.30–1.43)	1.25 (1.18–1.31)	<0.001
Average HU of Putamen	34.8 (32.2–27.1)	35.8 (34.3–38.0)	34.1 (31.3–36.2)	<0.001
Average HU of Corpus Callosum	27.0 (24.9–29.0)	26.4 (24.7–28.2)	27.2 (25.0–29.4)	0.002
Area ratio of the ventricle and the whole brain, Median (IQR)	0.110 (0.086–0.141)	0.108 (0.087–0.136)	0.110 (0.085–0.143)	0.449
Area of 2 lateral ventricles, ${\mathrm{mm}}^{2}$	7897 (6211–10,226)	7802 (6239–9947)	7963 (6195–10,298)	0.523
Area of the whole brain, ${\mathrm{mm}}^{2}$	73,095 (69,540–76,643)	73,351 (70,011–76,527)	72,727 (69,252–76,697)	0.480
Area of third ventricle, ${\mathrm{mm}}^{2}$	714 (524–993)	670 (500–926)	741 (552–1014)	0.057
Area of fourth ventricle, ${\mathrm{mm}}^{2}$	432 (331–561)	461 (380–617)	413 (313–545)	<0.001
Anteroposterior diameter of fourth ventricle, cm	0.89 (0.75–1.07)	0.95 (0.80–1.11)	0.86 (0.71–1.03)	<0.001
Abnormal MTLA (%)	26 (4.7)	7 (3.6)	19 (5.2)	0.520

Data presented as no. (%) or as median (IQR). HU, Hounsfield unit; GWR, grey-to-white matter ratio; MTLA, medial temporal lobe atrophy; IQR, interquartile range.

**Table 3.** **Characteristics of cerebral computed tomography between different severity groups based on sCAHP score**.

	Low severity	Moderate severity	High severity	*p*-value	*p* trend
GWR	1.339 (1.264–1.394)	1.293 (1.216–1.367)	1.253 (1.162–1.320)	<0.001	<0.001
Area ratio of the ventricle and the whole brain	0.104 (0.078–0.125)	0.127 (0.088–0.145)	0.117 (0.086–0.143)	0.008	0.111
Area of third ventricle, ${\mathrm{mm}}^{2}$	722.8 (491.0–902.0)	811.4 (548.3–1031)	783.7 (563.5–991.8)	0.064	0.137
Area of fourth ventricle, ${\mathrm{mm}}^{2}$	486.8 (364.0–627.0)	460.8 (345.3–549.3)	435.0 (307.3–532.3)	0.065	0.019
Anteroposterior diameter of fourth ventricle, cm	0.922 (0.770–1.090)	0.919 (0.800–1.070)	0.854 (0.700–1.023)	0.019	0.014
Abnormal MTLA (%)	9 (5.7)	11 (5.2)	3 (1.8)	0.170	0.089

Data presented as no. (%) or as median (IQR). GWR, grey-to-white matter ratio; MTLA, medial temporal lobe atrophy; IQR, interquartile range.

**Fig. 2.**
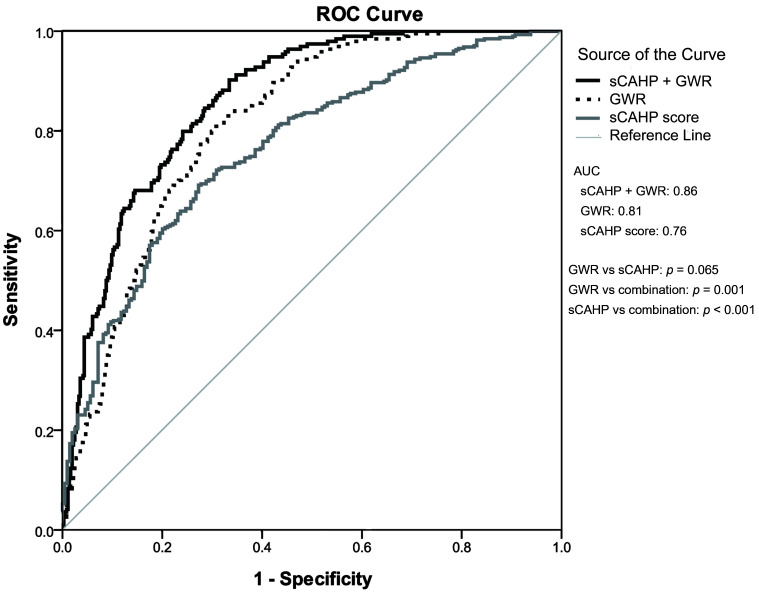
**The predictive performance of GWR and severity score for poor neurological outcome**. ROC, receiver operating characteristic; AUC, area under the receiver operating characteristic curve; GWR, gray-to-white ratio; sCAHP, simplified cardiac arrest hospital prognosis.

Table 4 presents the ability of GWR and sCAHP score in predicting neurological outcomes. The AUC was 0.81 (0.78–0.85) for GWR and 0.76 (0.72–0.80) for sCAHP score; no significant differences were noted (*p* = 0.065). The combination of GWR and sCAHP score exhibited significantly higher prognostication performance than either individual marker (GWR vs combination: *p* = 0.001; sCAHP vs combination: *p *< 0.001; Fig. 2) and exhibited greater predictive accuracy for subgroups of patients with OHCA, those with initial nonshockable rhythm, and those receiving TTM.

**Table 4.** **Predictive ability of GWR, severity score and their combination**.

Predictive marker		GWR	sCAHP	sCAHP + GWR
Overall		0.81 (0.78–0.85)*	0.76 (0.72–0.80)*	0.86 (0.83–0.89)
Subgroup				
	Age <65	0.85 (0.80–0.89)	0.73 (0.66–0.79)	0.86 (0.82–0.91)
	Age ≥65	0.80 (0.75–0.85)	0.77 (0.71–0.82)	0.85 (0.81–0.90)
	OHCA	0.85 (0.82–0.89)	0.80 (0.75–0.84)	0.90 (0.88–0.93)
	IHCA	0.73 (0.66–0.81)	0.71 (0.63–0.78)	0.79 (0.72–0.86)
	Nonshockable	0.83 (0.79–0.88)	0.79 (0.74–0.84)	0.88 (0.84–0.91)
	Shockable	0.79 (0.73–0.86)	0.71 (0.64–0.78)	0.84 (0.78–0.89)
	Non-TTM	0.81 (0.77–0.86)	0.74 (0.69–0.79)	0.85 (0.81–0.89)
	TTM	0.82 (0.76–0.87)	0.81 (0.75–0.87)	0.88 (0.84–0.92)

*p < 0.001 when compared with sCAHP + GWR. OHCA, out-of-hospital cardiac arrest; IHCA, in-hospital cardiac arrest; TTM, targeted temperature management; sCAHP, simplified cardiac arrest hospital prognosis; GWR, grey-to-white matter ratio.

## 4. Discussion

In this retrospective cohort study, we observed that cardiac arrest survivors with poor neurological recover were associated with lower GWR and fourth ventricle size. The GWR and fourth ventricle size decreased as postarrest severity increased in cardiac arrest survivors. Combining GWR with sCAHP score significantly improved predictive ability (vs either alone), particularly for patients with OHCA, those with initial nonshockable rhythm, and those receiving TTM.

Various neuroimaging tools have been developed for neuroprognostication. Several studies have validated GWR as a marker of neurological outcomes of cardiac arrest [11, 12, 13]. The Coronary Angiography After Cardiac Arrest trial included only patients with OHCA with initial shockable rhythm; the results of a post hoc analysis performed in this trial indicated GWR to be a poor neurological prognostic marker [31]. In our study, the GWR to have fair value for neuroprognostication. The difference in these results regarding the predictive ability of GWR might originate from differences in the inclusion criteria of the aforementioned study and ours. Very few studies have focused on neurological outcome prediction based on brain ventricle characteristics, and the reported results are diverse. Lee *et al*. [15] included cardiac arrest patients who underwent TTM and reported that a decrease in third ventricle area may indicate favorable neurological outcomes but observed no significant differences in the fourth ventricle area. Our findings suggested similar trends for third ventricle area, but statistical significance was not reached. Cerebral edema may affect the aqueduct connecting the third and fourth ventricles. Even mild edema narrows the aqueduct and cause an obstruction. The third ventricle may consequently increase in size. However, another study of OHCA patients who received therapeutic hypothermia reported no neuroprognostic value of third ventricle area [14], yet patients with larger fourth ventricle areas had more favorable neurological prognoses. Our study showed similar results. Well-designed studies are warranted to clarify these inconclusive results regarding third and fourth ventricle area. In summary, more severe brain edema appears to be associated with lower GWR; however, the correlation between fourth ventricle area and neurological outcomes requires further study. The VBR as well as MTLA scores were analyzed on the basis of the hypothesis that brain atrophy may complicate brain edema and intracranial pressure and thus influence neurological outcomes. However, no significant results were observed for any of these measurements.

GWR has been combined with other laboratory or clinical assessments, such as imaging [32, 33], electroencephalography [34], and blood tests [35], to improve its prognostic performance. Such combinations improved the prediction of neurological outcomes. Although various scoring systems based on medical history and CPR events have been developed, few studies have evaluated the performance of GWR in combination with a clinical scoring system for predicting neurological outcomes after cardiac arrest [25]. We evaluated the ability of the combination of GWR and sCAHP score for neuroprognostication, which was superior to that of either indicator alone. The CAST score, proposed by Nishikimi *et al*. [36], includes GWR and also exhibited good predictive ability (AUC = 0.971) in external validation.

In subgroup analysis, the predictions of GWR, sCAHP, and their combination were more accurate for patients with OHCA than for those with IHCA. These findings are consistent with those of previous studies. Yeh *et al*. [10] used GWR to predict survival and neurological outcomes in OHCA survivors and reported promising results. However, Ong *et al*. [37] reported no predictive power of GWR for survivors of IHCA. Carrick *et al*. [38] performed a systemic review of clinical predictive models of sudden cardiac arrest; predictive performance was better for patients with OHCA than for those with IHCA. This result might be due to patients with IHCA receiving immediate medical attention and advanced cardiac life support, unlike patients with OHCA; thus, hypoxic–ischemic brain injury was less severe among the patients with IHCA [10, 37, 38]. Therefore, GWR is more effective for predicting the neurological outcomes of OHCA survivors. Also need to be mentioned that sCAHP scores are more suitable for survivors of OHCA than of IHCA, since the sCAHP score is established on data from OHCA survivors [24].

## 5. Limitations

This study has some limitations. First, because of the retrospective nature, selection bias was unavoidable; moreover, unidentified confounding factors might have been present. Second, although the intraclass correlation coefficient was high, practical measurements of neuroimaging parameters may vary across raters. Third, 12 of the total 559 included patients received cardiac catheterization before the cerebral CT, and the contrast used in the coronary angiography may influence HU value and GWR in some case reports [39, 40]. Fourth, the Coma Recovery Scale-Revised (CRS-R) score may be more accurate than CPC scale in evaluating the neurological outcome of cardiac arrest survivors with disorder of consciousness [41]. However, due to the retrospective nature, some certain functional tests needed for the calculation, such as auditory or visual function, were not recorded. Finally, this study was conducted at a single tertiary medical center in an urban region; however, the protocol for the treatment and transport of patients with cardiac arrest may be different from those in rural regions or at primary care centers. Thus, in different clinical settings the prognostic scoring system should be applied with caution.

## 6. Conclusions

In cardiac arrest survivors, GWR and the size of the fourth ventricle were associated with neurological recovery. GWR as well as the area and anteroposterior diameter of the fourth ventricle decreased as postarrest severity increases. Combining GWR and sCAHP score may improve the ability of neuroprognostication.

## Data Availability

The datasets used and/or analyzed during the current study are available from the corresponding author on reasonable request.
